# Altered Fronto-Striatal Fiber Topography and Connectivity in Obsessive-Compulsive Disorder

**DOI:** 10.1371/journal.pone.0112075

**Published:** 2014-11-06

**Authors:** Takashi Nakamae, Yuki Sakai, Yoshinari Abe, Seiji Nishida, Kenji Fukui, Kei Yamada, Manabu Kubota, Damiaan Denys, Jin Narumoto

**Affiliations:** 1 Department of Psychiatry, Graduate School of Medical Science, Kyoto Prefectural University of Medicine, Kyoto, Japan; 2 Computational Neuroscience Laboratories, Advanced Telecommunications Research Institute International, Kyoto, Japan; 3 Department of Radiology, Graduate School of Medical Science Kyoto Prefectural University of Medicine, Kyoto, Japan; 4 Department of Psychiatry, Graduate School of Medicine, Kyoto University, Kyoto, Japan; 5 Department of Neuropsychiatry, Academic Medical Center, University of Amsterdam, Amsterdam, the Netherlands; 6 The Netherlands Institute for Neuroscience, an institute of the Royal Netherlands Academy of Arts and Sciences, Amsterdam, the Netherlands; West China Hospital of Sichuan University, China

## Abstract

Fronto-striatal circuits are hypothesized to be involved in the pathophysiology of obsessive-compulsive disorder (OCD). Within this circuitry, ventral frontal regions project fibers to the ventral striatum (VS) and dorsal frontal regions to the dorsal striatum. Resting state fMRI research has shown higher functional connectivity between the orbitofrontal cortex (OFC) and the dorsal part of the VS in OCD patients compared to healthy controls (HC). Therefore, we hypothesized that in OCD the OFC predominantly project fibers to the more dorsal part of the VS, and that the structural connectivity between the OFC and VS is higher compared to HC. A total of 20 non-medicated OCD patients and 20 HC underwent diffusion-weighted imaging. Connectivity-based parcellation analyses were performed with the striatum as seed region and the OFC, dorsolateral prefrontal cortex, and dorsal anterior cingulate cortex as target regions. Obtained connectivity maps for each frontal region of interest (ROI) were normalized into standard space, and *Z*-component (dorsal–ventral) coordinate of center-of-gravity (COG) were compared between two groups. Probabilistic tractography was performed to investigate diffusion indices of fibers between the striatum and frontal ROIs. COG *Z*-component coordinates of connectivity maps for OFC ROI were located in the more dorsal part of the VS in OCD patients compared to HC. Fractional anisotropy of fibers between the OFC and the striatum was higher in OCD patients compared to HC. Part of the pathophysiology of OCD might be understood by altered topography and structural connectivity of fibers between the OFC and the striatum.

## Introduction

Obsessive-compulsive disorder (OCD) is characterized by anxiety-provoking thoughts or images (obsessions) and repetitive behaviors that are performed to reduce anxiety (compulsions). Fronto-striatal circuits are hypothesized to be involved in the pathophysiology of OCD because both structural and functional neuroimaging research showed abnormalities in these circuits [1]–[5]. In particular, resting state functional MRI research consistently reported higher functional connectivity between the frontal and the striatum regions in OCD compared to healthy controls (HC) [6]–[11] except for one report [12].

Within this circuitry, the ventral prefrontal cortex (vPFC) including orbitofrontal cortex (OFC) mainly projects fibers to the ventral striatum, while the dorsal frontal cortex such as dorsolateral prefrontal cortex (DLPFC) and dorsal anterior cingulate cortex (dACC) project to the dorsal striatum [13]–[15]. This specific topography within the circuitry was originally shown by tracer studies in non-human primates [15]. Then, it was replicated in human brain using connectivity-based parcellation analysis [14]. Connectivity-based parcellation is a new analysis of probabilistic tractography on diffusion weighted imaging (DWI) data, which was originally used to segment the thalamus [16]. Probabilistic tractography is able to trace these fibers in vivo and discriminate human thalamic subregions on the basis of their connections with the cortex. The segmented subregions accurately corresponded to the thalamic nuclei that were previously described in histological studies. Thus, connectivity-based parcellation analysis on DWI data is accurate enough to describe in vivo human fiber connection and anatomical structures. This reliable method has been applied to segment the striatum based on its connectivity to the frontal areas, and the above-mentioned specific topography has been replicated repeatedly [14], [17], [18]. In addition, Johansen-Berg et al. [19] reported that connectivity-based parcellation analysis could detect individual variation of fiber topography even in healthy subjects. It is therefore possible to detect difference in fiber topography between patients and controls.

Our previous resting state fMRI research showed higher functional connectivity between the dorsal part of the ventral striatum and the OFC in OCD patients compared to HC while there was no significant change of functional connectivity between the ventral part of the ventral striatum and the OFC versus HC [8]. Therefore, we hypothesized that the fiber topography of the fronto-striatal circuits is altered in OCD patients, and that the OFC projects predominantly fibers to the more dorsal part of the striatum compared to HC. The first aim of our study is thus to compare the fiber topography of the circuits in OCD and HC by using connectivity-based parcellation analysis to verify our hypothesis.

Additionally, we hypothesized that patients with OCD also show higher structural connectivity between the OFC and the ventral striatum compared to HC. Structural connectivity represents the anatomical white-matter connection between regions, which depends on degree of myelination and number of fibers, while functional connectivity refers to the synchrony between a continuous time series of brain activity (Table 1). In general, functional connectivity reflects structural connectivity because the stronger brain regions are anatomically connected the more efficiently they can communicate [20]. The structural connectivity can be inferred using DWI. Although fractional anisotropy (FA) is the most commonly used DWI index, it does not directly reflect structural connectivity as it is modulated not only by degree of myelination and number of fibers but also by membrane permeability and fiber orientation in each voxel [21]. On the other hand, other diffusion indices such as mean diffusivity (MD), axial diffusivity (AD), and radial diffusivity (RD) give more detailed information about the biological processes that underlie the observed or undetectable changes in FA. Especially, RD reflects water mobility perpendicular to the fiber axis. Higher structural connectivity by increasing axonal density and the degree of myelination would lead to reduced RD and increased FA [21]. Previous DWI studies in OCD have shown abnormalities of white matter tracts, including the corpus callosum, anterior limb of the internal capsule, and cingulum [22]–[24]. However, the results are inconsistent due to methodological shortcomings such as bias by medication treatment, which may impact the results [25],[26], or methodological problem including misregistration and smoothing when voxel-based analysis is applied for DWI data [27]. No studies have directly investigated topography and connectivity of fibers between the frontal regions and the striatum using tractography. Since conventional single tensor tractography is inadequate to reconstruct fibers correctly, and a deterministic approach is not able to depict branching fibers because it produces one reconstructed trajectory per seed point [21], [28], probabilistic tractography is the technique of choice to explore structural connectivity. Therefore, the second aim of this study is to directly investigate white matter structural connectivity between the frontal regions and the striatum in non-medicated patients with OCD using probabilistic tractography. In particular, we predicted that higher FA concurrent with lower RD that corresponds to higher structural connectivity might be found in the fibers between the OFC and the striatum as we hypothesized above.

**Table 1:** Explanation of connectivity and topography.

	Modality	Index	Biological Implications
Functional connectivity	resting state fMRI	Correlation coefficient of BOLD signalfluctuation	Synchronization of brain activities betweenseed and target regions
Structural connectivity	Probabilistic tractography on DWI	Fractional anisotropy and radialdiffusivity of reconstructed fibers*	Degree of myelination and number of fibersbetween seed and target regions
Fiber topography	Connectivity-based parcellation on DWI	COG of connectivity map	Distribution of fibers’ projection fromseed to target regions

BOLD: blood oxygen level-dependent, COG: center-of-gravity, DWI: diffusion weighted imaging.

*Note that fractional anisotropy does not exactly represent strength of structural connectivity because it is modulated not only by degree of myelination and number of fibers but also by membrane permeability and fiber orientation in each voxel.

## Methods and Materials

### Participants

The subjects were 20 adult patients (9 men and 11 women) diagnosed with OCD (based on the DSM-IV criteria) and 20 healthy volunteers (6 men and 14 women) matched for age and handedness. Patients were recruited at the Kyoto Prefectural University of Medicine Hospital, Kyoto, Japan. All patients were primarily diagnosed using the Structured Clinical Interview for DSM-IV Axis I Disorders-Patient Edition (SCID) [29]. All patients had a sole diagnosis of OCD and none had been taking any kind of psychotropic medication for at least 8 weeks, of which 5 were drug naïve. All patients were tested with the Yale-Brown Obsessive-Compulsive Scale (Y-BOCS) to assess the severity of OCD symptoms [30], the 17-item Hamilton Depression Rating Scale (HDRS) to assess the severity of depression [31], and the Hamilton Anxiety Rating Scale (HARS) to assess the severity of anxiety [32], Patients who had full remission were excluded (defined as a Y-BOCS score <10) [33]. Exclusion criteria for patients and healthy volunteers were: 1) significant disease such as neurological diseases, pulmonary, cardiac, renal, hepatic, endocrine systems, and metabolic disorders; 2) current or past DSM-IV axis I diagnosis of any psychiatric illness except OCD; and 3) DSM-IV diagnosis of mental retardation and pervasive developmental disorders based on a clinical interview and psychosocial history. There was no history of psychiatric illness in the healthy volunteers as determined by the Structured Clinical Interview for DSM-IV Axis I Disorders-Non-patient Edition (SCID-NP). In addition, we confirmed no psychiatric treatment history in any of the healthy volunteers’ first-degree relatives. Kyoto Prefectural University of Medicine Research Ethics Committee approved all procedures. All participants gave written, informed consent after receiving a complete description of the study.

### MRI Acquisition and Preprocessing

High-resolution T1-weighted and diffusion-weighted images (DWIs) were obtained with a whole-body 3-Tesla MR system (Achieva 3.0 TX; Philips Medical Systems, Best, The Netherlands) with an eight-channel phased-array head coil. T1-weighted images were used to define anatomically correct regions of interests based on cortical and subcortical structures as described below. A combination of T1-weighted images and DWIs is therefore advantageous to delineate specific fibers within a neural circuitry. The scanning parameters of the T1-weighted three-dimensional magnetization-prepared rapid gradient-echo (3D-MPRAGE) sequences were as follows: flip angle, 10 degrees; acquisition matrix, 256×256×170; field of view, 25.6 cm; section thickness, 1.0 mm; voxel size, 1.0 mm×1.0 mm×1.0 mm; TR, 7.1 ms; and TE, 3.3 ms. The 3D-MPRAGE images were preprocessed using the FreeSurfer software package version 5.2.0 (http://surfer.nmr.mgh.harvard.edu). [34], [35] In brief, the processing stream included affine transformation of each subject’s native brain to the MNI305 atlas, removal of non-brain tissue, volumetric subcortical labeling [36], and surface based segmentation of grey matter and white matter tissue [37],[38].

Diffusion-weighted data were obtained using a single-shot spin-echo echo-planar sequence with 32 Stejskal–Tanner motion-probing gradient orientations. DWIs protocol were as follows: b = 1000 s/mm^2^; 60 axial slices with 2.0 mm thickness without gap; TR, 7181 ms, TE, 58 ms, flip angle, 90 degrees; 112×110 matrix; field of view, 224×224×120 mm; voxel size = 2×2×2 mm. One b = 0 image was obtained for each subject therefore each subject had 33 volumes. DWIs data processing was performed using programs in the FMRIB Software Library (FSL) version 5.0.2 (http://www.fmrib.ox.ac.uk/fsl). All DWIs source data were corrected for eddy currents and head motion by registering each data point to the first b = 0 image with affine transformation. The FA, MD, AD, and RD maps were calculated using the DTIFIT program implemented in FSL. In order to perform connectivity-based parcellation and probabilistic tractography, probability distributions on 2 fiber directions were modeled at each voxel using FSL’s BedpostX program, based on a multifiber diffusion model [39]. A board-certified neuroradiologist reviewed all scans and found no gross abnormalities in any of the subjects.

### Regions of Interests (ROIs) definition

**Figure 1:**
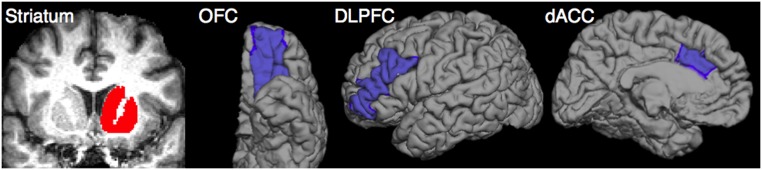
The striatum and 3 frontal ROIs (OFC, DLFPC, and dACC) in the left hemisphere.

The striatum ROIs were created with a combination of caudate, putamen, and nucleus accumbens that were extracted from the automated segmentation in FreeSurfer [36]. The OFC, DLPFC, and dACC were chosen as frontal ROIs because they are the most relevant frontal regions in the pathophysiology of OCD. They were also extracted from the surface-based procedure in FreeSurfer, based on cortical parcellation with Desikan–Killiany–Tourville protocol [40]. The ROIs of OFC, DLFPC, and dACC were defined by the lateral orbitofrontal, rostral middle frontal, and caudal anterior cingulate labels respectively (Fig. 1).

### Connectivity-Based Parcellation

**Figure 2:**
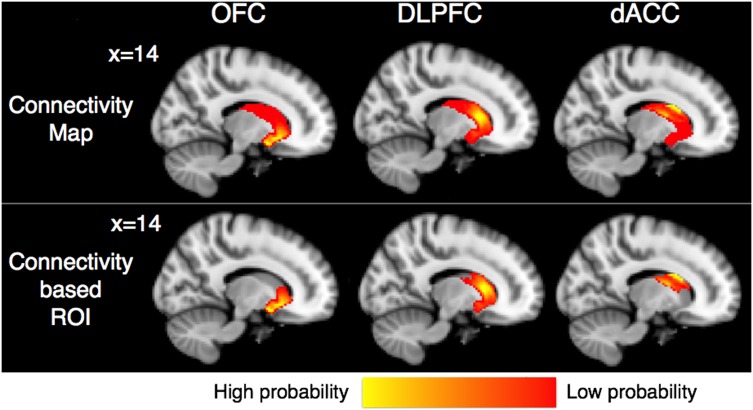
Mean images of connectivity maps and connectivity-based ROIs of the striatum for each frontal ROI (OFC, DLPFC, and dACC) are shown on the same sagittal section (*X* = 14) in MNI152 standard space. Images of all subjects from both HC and OCD groups are averaged and only the voxels where at least 10 of 40 subjects’ data are overlapped are shown. The ventral striatum had high probabilities of connection to OFC, while dorsal striatum had high probable connection with DLPFC and dACC. Connectivity-based ROIs are created by thresholding connectivity maps at 25% and used as the target ROIs in following probabilistic tractography analysis.

Each subject’s b = 0 image was co-registered to a T1 image by applying the rigid transformation matrix, which was calculated by use of FSL’s FLIRT program [41]. Then, the obtained matrix was inversed using “convert_xfm” command to transform images from 3D-MPRAGE space to diffusion space. The striatum and the 3 frontal ROIs were transformed from each subjects’ 3D-MPRAGE space to diffusion space. To check the quality of the transformation, we visually inspected each mask in the diffusion space for each subject and confirmed that there were no gross transformation errors. Connectivity-based parcellation analyses were performed in diffusion space with seed region as the striatum and target regions as OFC, DLPFC, and dACC using FSL’s BedpostX program based on a multifiber diffusion model, separately for each hemisphere. Streamlined samples were traced through the probabilistic distributions of fiber direction, with 5000 iterations per striatum seed voxel (curvature thresholds, 0.2). Then, obtained connectivity maps for each frontal ROI were transformed from each subjects’ 3D-MPRAGE space into Montréal Neurological Institute (MNI) 152 standard space by applying the non-linear transformation matrix, which was calculated by use of FSL’s FNIRT program (Fig. 2). Center-of-gravity (COG) of spatially normalized connectivity maps was calculated using “cluster” command implemented in FSL (http://fsl.fmrib.ox.ac.uk/fsl/fslwiki/Cluster). In detail, we used following command line.

cluster – in = <each connectivity map> – thresh = 0 – mm.

Here, – in, – thresh, and – mm arguments represent input file, threshold, and use millimeter (not voxel coordinates), respectively. COG was used as an index of fiber spatial distribution as it is used in previous connectivity-based parcellation studies [42], [43]. The *Z*-component (dorsal–ventral) COG of spatially normalized connectivity maps were identified from above “cluster” command line. Then, they were compared between the two groups by analysis of covariance (ANCOVA) controlled for age and gender effects because both of them might affect white matter structure [44], [45]. SPSS version 21.0 was used for these analyses, and statistical significance were defined as *P*<.05 (2-tailed; not corrected for multiple comparisons).

### Probabilistic Tractography

The same 3 frontal ROIs were used as seed ROIs in probabilistic tractography. Each subject’s connectivity maps for each frontal ROI was thresholded at 25% of the maximum connectivity probabilities to exclude voxels with low connectivity probabilities. Thus, connectivity-based striatum ROIs were created and used as the target ROIs (Fig. 2, Fig. S1). This threshold was decided based on previous studies [19], [46], [47]. These ROIs were transformed from each subjects’ 3D-MPRAGE space to diffusion space using the above inversed matrix. Probabilistic tractography from the seed region (the OFC, DLPFC, and dACC ROI) to the target regions (the connectivity-based striatum ROI for each frontal ROI) was performed using FSL’s ProbtrackX program, separately for each hemisphere. Streamlined samples were traced through the probabilistic distributions of fiber direction, with 5000 iterations per striatum seed voxel (curvature thresholds, 0.2). To investigate only direct pathways from the frontal regions to the striatum, an exclusion mask was also created using an automated procedure in FreeSurfer. The exclusion mask consisted of cortical regions except the seed cortex, and all brain regions in the other hemisphere. Each tract was created in the each subjects’ 3D-MPRAGE space and thresholded to exclude voxels in which the streamlined sample count corresponded to the lower 15% of the outer tail of the histogram, to eliminate extraneous tracking results. The thresholded tracts were transformed back into diffusion space, and the mean diffusion indices including FA, MD, AD and RD values of each tract were calculated. Then, they were compared between the two groups by ANCOVA controlled for age and gender effects because both of them might affect white matter structure [44], [45]. SPSS version 21.0 was used for these analyses, and statistical significance was defined as *P*<.05 (2-tailed; not corrected for multiple comparisons).

### Regression and Correlation Analysis

In the patient group, multiple regression analyses were performed using each DWI index (FA, MD, AD, and RD) as a dependent variable and the Y-BOCS, HDRS and HARS total scores as independent variables to examine whether abnormalities of the DWI indices in patients with OCD are state or trait markers. We also examined Spearman rank-order correlations between age of onset, duration of illness, and *Z*-component coordinates of COG for each connectivity map. The statistical significance level was set at *P*<.05. Multiple comparisons were not corrected in this analysis. Data were analyzed using SPSS 21.0.

## Results

### Demographic Data

The demographic and clinical data are shown in Table 2. Age, sex, handedness, and education level did not differ significantly between patients and HC.

**Table 2:** Demographic and clinical characteristics of subjects in the healthy control and patient groups.

Characteristic	Healthy Controls (n = 20)	Patients with OCD (n = 20)	*P* value
Age, years	32.9±6.9	35.3±9.4	.363‡
Sex, male/female	9/11	6/14	.514†
Handedness, right/left	19/1	18/2	1.000†
Education, years	15.1±1.9	14.1±2.2	.133‡
Age of onset, years	NA	25.0±9.5	NA
Duration of illness, years	NA	10.3±6.5	NA
Psychotropic medication naïve/free patients	no medication	5/15	NA
Length of past medication, months	NA	20.2±26.5	NA
Total Y-BOCS score	NA	22.8±5.8	NA
HDRS score	NA	3.8±3.1	NA
HARS score	NA	5.3±4.5	NA

Abbreviations: HARS, Hamilton Anxiety Rating Scale; HDRS, Hamilton Depression Rating Scale; NA, not applicable; OCD, obsessive-compulsive disorder; Y-BOCS, Yale-Brown Obsessive-Compulsive Scale.

Values represent the mean ± SD. For all scales, high scores denote greater severity.

† *χ^2^* test.

‡ Independent sample *t* test.

### Connectivity-Based Parcellation

**Figure 3:**
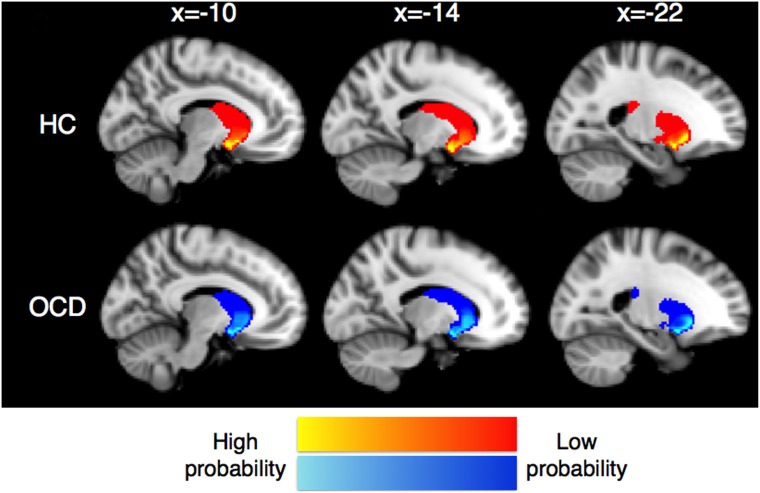
Mean images of connectivity maps for OFC ROI in HC (red-yellow) and OCD (blue-light blue) are shown in MNI152 standard space. Images of all subjects in each group are averaged and only the voxels where at least 5 of 20 subjects’ data are overlapped are shown. The ventral striatum showed high probabilities of connection with the OFC in both groups. However, the regions with high probabilities spread into more dorsal part of the striatum in OCD compared to HC group.

Mean images of connectivity maps for each frontal ROI and examples of some subjects’ results are shown in MNI152 standard space (Fig. 2) and each subject’s 3D-MPRAGE space (Fig. S1) respectively. The ventral striatum had high probabilities of connection to OFC, while the dorsal striatum had strong connectivity with DLPFC and dACC. The regions with high probabilities of connection with the OFC spread into more dorsal part of the striatum in patients with OCD compared to HC (Fig. 3). Mean COG *Z*-component coordinate of each connectivity map in two groups are shown in Table 3. COG *Z*-component coordinates of the connectivity maps for the left OFC were significantly larger in patients with OCD than those of HC (*P* = .043). Patients with OCD also showed marginally significant larger *Z*-component coordinates of the connectivity maps for the right OFC (*P* = .056). That is, they were located in the more dorsal area of the striatum in patients compared to HC.

**Table 3:** *Z*-component (dorsal-ventral) coordinates of center-of-gravity (COG) for each connectivity map in the MNI152 standard space.

		*Z*-component coordinate of COG (mm) Mean (SD)		
Seed ROI	Target ROI	Healthy Controls (n = 20)	Patients with OCD (n = 20)	*P* value†
Left striatum	OFC	−6.21±1.71	−5.08±1.71	.043*
	DLPFC	1.86±2.78	1.48±2.16	.649
	dACC	7.11±2.33	7.41±1.92	.573
Right striatum	OFC	−6.20±1.67	−5.21±1.78	.056
	DLPFC	3.14±2.64	3.25±3.01	.659
	dACC	10.05±4.05	9.60±4.56	.574

Abbreviations: dACC, dorsal anterior cingulate cortex; DLPFC, dorsolateral prefrontal cortex; OCD, obsessive-compulsive disorder; OFC, orbitofrontal cortex; ROI, region of interest.

† Analysis of covariance controlling for age and gender effects.

*P<.05 (not corrected for multiple comparisons).

### Probabilistic Tractography

**Figure 4:**
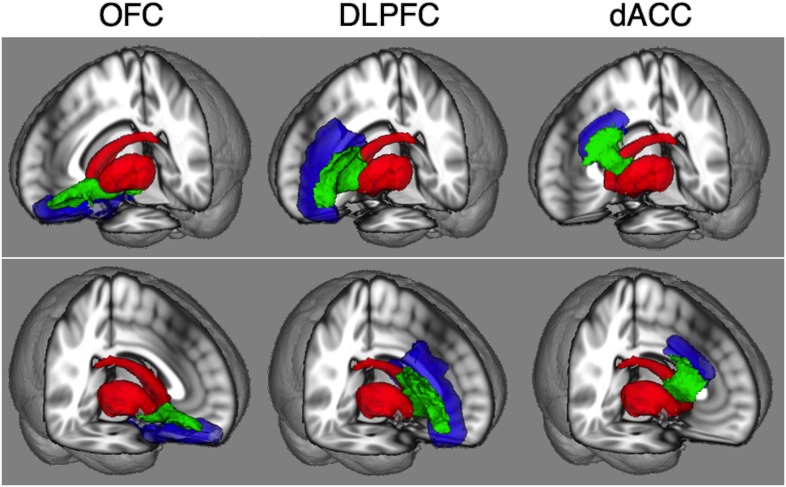
Mean images of probabilistic tractography results (green) that delineate fibers between the each frontal (blue) and striatum (red) regions are shown in MNI152 standard space. Images of all subjects from both HC and OCD groups are averaged and only the voxels where at least 10 of 40 subjects’ data are overlapped are shown.

Mean image of tractography results and examples of some subjects’ results are shown in MNI152 standard space (Fig. 4) and each subject’s diffusion space (Fig. S2) respectively. Mean FA values of fibers between each frontal region and the striatum are shown in Table 4. FA values of fibers between the left OFC and the striatum showed significantly higher in patients with OCD compared to HC (*P* = .025). Patients with OCD also showed a trend for higher FA of fibers between the right OFC and the striatum compared to HC (*P* = .062). The results of all diffusion indices for fibers between the OFC and the striatum are shown in Table 5. RD values of fibers between the left OFC and the striatum were also significantly lower than those of HC (*P* = .043). Other results are shown in Table S1 and S2. There were trends for higher FA (*P* = .091) of the left DLPFC-striatum fibers, for higher MD (*P* = .074) and higher AD (*P* = .061) of the right DLPFC-striatum fibers, and for higher FA of the right dACC-striatum fibers (*P* = .070) in OCD compared to HC.

**Table 4:** Fractional anisotropy value of fibers between each frontal region and the striatum in two groups.

	FA value, Mean (SD)		
Fiber	Healthy Controls (n = 20)	Patients with OCD (n = 20)	*P* value†
L OFC-Striatum	0.32±0.03	0.34±0.03	.025*
L DLPFC-Striatum	0.32±0.02	0.34±0.02	.091
L dACC-Striatum	0.37±0.04	0.38±0.04	.353
R OFC-Striatum	0.32±0.02	0.34±0.03	.062
R DLPFC-Striatum	0.33±0.02	0.32±0.02	.812
R dACC-Striatum	0.35±0.03	0.36±0.03	.070

Abbreviations: dACC, dorsal anterior cingulate cortex; DLPFC, dorsolateral prefrontal cortex; FA, fractional anisotropy; L, left; OCD, obsessive-compulsive disorder; OFC, orbitofrontal cortex; R, right.

† Analysis of covariance controlling for age and gender effects.

*P<.05 (not corrected for multiple comparisons).

**Table 5:** Diffusion indices of fibers between the OFC and the striatum.

Fibers	Diffusion indices	Healthy Controls (n = 20)	Patients with OCD (n = 20)	*P* value†
L OFC-Striatum	FA	0.32±0.03	0.34±0.03	.025*
	MD‡	0.81±0.03	0.79±0.03	.160
	AD‡	1.11±0.06	1.11±0.05	.901
	RD‡	0.66±0.04	0.64±0.03	.043*
R OFC-Striatum	FA	0.32±0.02	0.34±0.03	.062
	MD‡	0.80±0.03	0.81±0.04	.631
	AD‡	1.09±0.05	1.12±0.06	.180
	RD‡	0.66±0.04	0.66±0.05	.790

Abbreviations: AD, axial diffusivity; FA, fractional anisotropy; L, left; MD, mean diffusivity; OCD, obsessive-compulsive disorder; OFC, orbitofrontal cortex; R, right; RD, radial diffusivity.

† Analysis of covariance controlling for age and gender effects.

*P<.05 (not corrected for multiple comparisons).

‡ units = ×10^−3^ mm^2^/s.

### Regression and Correlation Analysis

None of the Y-BOCS, HDRS, and HARS total scores did predict DWI indices of fibers (Table S3). Age of onset showed a significantly negative correlation with Z-component coordinates of COG for almost all connectivity maps, but duration of illness did not (Table 6).

**Table 6:** Correlational analyses between age of onset, duration of illness, and Z-component coordinate of COG for each connectivity map in the patient group (N = 20).

Connectivity Maps		Age of Onset		Duration of Illness	
Seed ROI	Target ROI	Spearman’s *r*	*P* value	Spearman’s *r*	*P* value
L Striatum	OFC	−.442	.051	.044	.855
	DLPFC	−.469	.037*	−.290	.214
	dACC	−.459	.042*	−.002	.995
R Striatum	OFC	−.508	.022*	−.045	.850
	DLPFC	−.639	.002*	.079	.741
	dACC	−.481	.032*	.252	.284

*P<.05 (not corrected for multiple comparisons).

## Discussion

This is the first connectivity-based parcellation and probabilistic tractography study examining directly the fronto-striatal fiber topography and structural connectivity of non-medicated patients with OCD and matched HC. We found dorsally spreading projection of fibers between the OFC and the striatum in patients with OCD, as we hypothesized, and higher FAs of these fibers in patients with OCD compared to HC based on the connectivity-based parcellation and the probabilistic tractography analyses respectively.

Previous DWI studies in OCD have shown abnormalities of white matter tracts, including the corpus callosum, anterior limb of the internal capsule, and cingulum [24]. However, most of previous DWI studies applied conventional voxel-based analysis or tract-based spatial statistics. As large fiber tracts contain several kinds of fibers that connect to different regions, abnormalities detected in these large fibers might refer to all of the fiber tracts included. Therefore, DWI studies that investigate which specific fibers are related to the pathophysiology of OCD should be conducted. This is the first study that directly investigated topography and connectivity of fibers between the frontal and the striatum.

Connectivity-based parcellation analyses showed dorsally spreading projection of fibers between the OFC and the striatum in patients with OCD, which is consistent with a finding of our previous resting state fMRI research that showed higher functional connectivity between the dorsal part of the ventral striatum and the OFC [8].

In addition, probabilistic tractography analyses showed the higher FAs of fibers between the OFC and the striatum. It can be affected by several factors such as degree of myelination, number of fibers, membrane permeability, and fiber orientation in each voxel. However, the left OFC-striatum fibers also showed lower RD that reflects water mobility perpendicular to the fiber axis. Myelination could decrease RD by increasing the barriers for water molecular diffusion perpendicular to the myelinated axons [48]. Therefore, higher FA concurrent with lower RD might represent increased myelination of these fibers. Thus, a higher FA of fibers between the OFC and the striatum might represent hyper structural connectivity between these regions. Previous multimodal neuroimaging researches have shown positive correlation between FA value and functional connectivity between two brain regions [20]. Therefore, hyper structural connectivity between the OFC and the striatum is consistent with hyper functional connectivity between these regions as shown by previous resting state fMRI research [7]–[10]. In addition, optogenetic animal research showed that hyperactivity between the OFC and the ventral striatum might cause repetitive behavior [49]. Repeated and chronic hyper activation of fibers over multiple days generated OCD-like grooming behavior, which persisted for 2 weeks after stimulation cessation while acute OFC-striatum stimulation did not produce repetitive behaviors. It is therefore plausible that a specific and minimum period of hyper activation might cause long-lasting changes including synaptic plasticity or white matter structural connectivity. This is substantiated by other optogenetic research. It was shown Sapap3 knockout mice that selective stimulation of the lateral OFC-striatal pathways prevents overexpression of both conditioned and spontaneous repetitive behavior [50]. Our results are consistent with these findings since it confirms that fibers between the OFC and the striatum are involved in the pathophysiology of OCD.

From both connectivity-based parcellation and probabilistic tractography results, we did not find any topographical and connectivity abnormalities in fibers between the DLPFC, dACC and the striatum except for trends for higher FA of the left DLPFC-striatum fibers, higher MD and higher AD of the right DLPFC-striatum fibers, and higher FA of the right dACC-striatum fibers in OCD compared to HC. These findings were only found in the unilateral hemisphere, though we did not have a hypothesis for these fibers’ abnormalities. Therefore, further studies will be necessary to confirm the involvement of white matter abnormalities in the fibers between the DLPFC, dACC and the striatum in patients with OCD.

Multiple regression analyses showed that severity of OCD, of comorbid depression and anxiety did not predict DWI indices. A higher FA or lower RD in patients with OCD compared to HC probably represents a trait rather than a state marker. However, the results should be interpreted with extreme caution because of the small sample size of this study. This finding could be clarified by examining first-degree relatives of patients with OCD. Age of onset, but not disease duration showed a significant negative correlation with Z-component coordinates of COG for almost all connectivity maps. This finding hints to the hypothesis that abnormalities of fiber topography may be related to the specific pathophysiology of early-onset OCD, which could be examined by comparing early with late-onset-patients with OCD.

Although the fiber topography and connectivity develop over time, it is unknown whether abnormalities of white matter structure are causally related to the etiology of OCD. FA values of white matter in healthy subjects increase during their childhood and adolescents [51], [52]. Previous studies reported higher FA and hyperconnectivity of various brain regions in child and adolescent OCD patients compared to healthy controls [53], [54] and suggested premature myelination hypothesis. On the other hands, FA values of frontostriatal fibers decrease after 20 years of age [44]. Given that the mean age of our sample was approximately 35 years and that patients showed higher FA of fibers between the OFC and the striatum compared to healthy controls, age-related decreases in FA values of the fibers in patients with OCD might be different from those of healthy controls.

Although all patients with OCD were non-medicated, medication could have affected the fronto-striatal fiber structure. We compared fiber topography and connectivity between two groups covarying not only age and gender but also the length of previous medication. The results remained unchanged (see Tables S4–S7). This finding is consistent with a previous paper that showed no significant difference in DWI indices between drug naïve and previously medicated patients with OCD [55].

Our findings from connectivity-based parcellation analysis might be related to clinical application. Dorsally spreading projection of fibers between the OFC and the striatum in patients with OCD might be particularly relevant to optimization of deep brain stimulation (DBS) settings since it targets the ventral striatum. DBS is the most promising treatment for refractory OCD patients although its mechanism is still unclear. Nucleus accumbens DBS seems to affect not only the nucleus accumbens itself but also the adjacent fibers because the active contact points of DBS effective in reducing anxiety and OCD symptoms were located near the internal capsule [56]. Lehman et al. [57] showed with tracing and tractography-based 3D reconstructions of white matter tracts in non-human primate how vPFC projections reach their targets. They showed that the topography of fibers from the vPFC is preserved in the human brain [58]. Subcortical fibers from each vPFC region travel dorsally to the brainstem with considerable differences in fibers, likely to be modulated differently by DBS within the same surgical target. So, though DBS targets one single region, it may affect different regions via different fiber bundles, necessitating information of fiber topography to optimize the DBS settings. If stimulation of fibers between the OFC and the ventral striatum are related to improvement of OCD symptoms, volume of tissue activated by DBS should include the dorsal part of the ventral striatum because fibers from the OFC spread more dorsally in OCD patients compared to HC. Further research will be needed to investigate the relationship between fronto-striatal fiber topography and optimal DBS settings in refractory OCD patients.

This study has several limitations. The first one is that we used the MNI305 atlas to transform original T1 images into standard space in the Freesurfer analysis. The atlas was not representative of our samples. This could be a limitation of the study although this method is validated and used as default setting in Freesurfer analysis [59]. Secondly, we did not include the ventromedial prefrontal cortex in the analysis, although this region is known to be important in the pathophysiology of OCD. It was excluded because this region tends to be affected by the susceptibility artifact because of air-filled sinuses. Field-map correction should be performed to correct susceptibility artifacts in future studies. The third limitation is that the patients included in this study had mild to moderate OCD symptoms. On the other hand, they were all non-medicated, excluding the effect of psychotropic medication. The fourth limitation is that we did not correct for multiple comparisons since we had a priori hypotheses for abnormalities of topography and connectivity of the fibers between the OFC and the striatum. We run however the risk of finding false positives, and therefore our results should be interpreted with caution. Finally, FA does not exactly represent strength of structural connectivity because it is modulated by not only degree of myelination and number of fibers but also membrane permeability and fiber orientation in each voxel [21]. New methods such as myelination imaging should be combined to improve assessment of structural connectivity [60].

In conclusion, our hypothesis was supported by the connectivity-based parcellation analysis. That is, the OFC predominantly project fibers to the more dorsal part of the striatum in patients with OCD compared to HC. This study also showed hyper structural connectivity of fibers between the OFC and the striatum. Part of the pathophysiology of OCD might be understood by altered topography and structural connectivity of fibers between the OFC and the striatum.

## Supporting Information

Figure S1 **Randomly selected examples of the connectivity maps (left panel) and connectivity-based ROIs (right panel) for each frontal ROI (OFC, DLPFC, and dACC) in the 3D-MPRAGE space.** Yellow represent high probabilities of structural connection with each ROI, while red represents low probabilities. The ventral striatum had high probabilities of connection to the OFC, while the dorsal striatum had high probable connection with the DLPFC and dACC. Connectivity-based ROIs were created by thresholding connectivity maps at 25% and used as the target ROIs in following probabilistic tractography analysis.
(JPG)

Figure S2 **Randomly selected examples of tractography results.** The frontal (blue), striatum (red) regions, and delineated fibers (green) were shown in diffusion space rendered on each subject’s fractional anisotropy map. Fibers between the OFC, DLPFC and the striatum were shown in axial section, while fibers between the dACC and the striatum were shown in coronal section. Images are shown with and without the ROIs and fibers to clarify the locations. Left-right orientation is according to radiological convention.
(JPG)

Table S1 **Diffusion indices of fibers between the DLPFC and the striatum.**
(DOC)

Table S2 **Diffusion indices of fibers between the dACC and the striatum.**
(DOC)

Table S3 **Results of multiple regression analyses predicting each DWI index from the Y-BOCS, HDRS and HARS total scores in patients with OCD (N = 20,** ***df*** **= 16).**
(DOC)

Table S4 ***Z*** **-component (dorsal-ventral) coordinates of center-of-gravity (COG) for each connectivity map in the MNI152 standard space controlling for past medication effects.**
(DOC)

Table S5 **Diffusion indices of fibers between the OFC and the striatum controlling for past medication effects.**
(DOC)

Table S6 **Diffusion indices of fibers between the DLPFC and the striatum controlling for past medication effects.**
(DOC)

Table S7 **Diffusion indices of fibers between the dACC and the striatum controlling for past medication effects.**
(DOC)
