# Supplementary figures and images for: Altered Fronto-Striatal Fiber Topography and Connectivity in Obsessive-Compulsive Disorder
Source: PLoS One. 2014 Nov 6;9(11):e112075. doi: 10.1371/journal.pone.0112075 (PMC4222976; doi:10.1371/journal.pone.0112075)

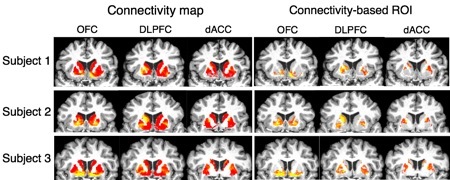

Supplement: Figure S1 — Randomly selected examples of the connectivity maps (left panel) and connectivity-based ROIs (right panel) for each frontal ROI (OFC, DLPFC, and dACC) in the 3D-MPRAGE space. Yellow represent high probabilities of structural connection with each ROI, while red represents low probabilities. The ventral striatum had high probabilities of connection to the OFC, while the dorsal striatum had high probable connection with the DLPFC and dACC. Connectivity-based ROIs were created by thresholding connectivity maps at 25% and used as the target ROIs in following probabilistic tractography analysis. (JPG) [file pone.0112075.s001.jpg]

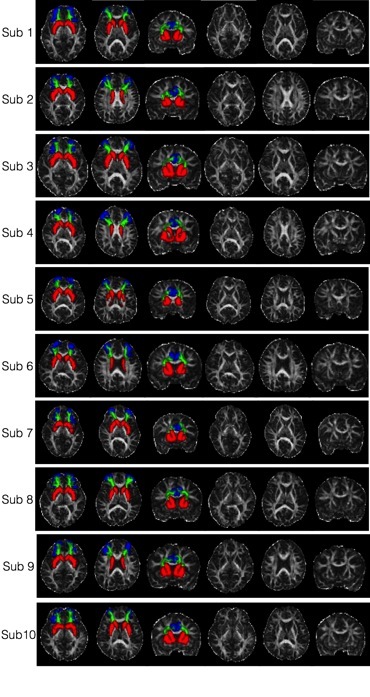

Supplement: Figure S2 — Randomly selected examples of tractography results. The frontal (blue), striatum (red) regions, and delineated fibers (green) were shown in diffusion space rendered on each subject’s fractional anisotropy map. Fibers between the OFC, DLPFC and the striatum were shown in axial section, while fibers between the dACC and the striatum were shown in coronal section. Images are shown with and without the ROIs and fibers to clarify the locations. Left-right orientation is according to radiological convention. (JPG) [file pone.0112075.s002.jpg]
