# Supplementary material for: Altered Fronto-Striatal Fiber Topography and Connectivity in Obsessive-Compulsive Disorder
Source: PLoS One. 2014 Nov 6;9(11):e112075. doi: 10.1371/journal.pone.0112075 (PMC4222976; doi:10.1371/journal.pone.0112075)
Supplement: Table S1 — Diffusion indices of fibers between the DLPFC and the striatum. (DOC) [file pone.0112075.s003.doc]

**Table S1.** Diffusion indices of fibers between the DLPFC and the striatum

| **Fibers** | **Diffusion indices** | **Healthy Controls (n = 20)** | **Patients with OCD (n = 20)** | ***P* value†** |
| --- | --- | --- | --- | --- |
| L DLPFC-Striatum | FA | 0.32 ± 0.02 | 0.34 ± 0.02 | .091 |
|  | MD‡ | 0.77 ± 0.03 | 0.77 ± 0.02 | 1.000 |
|  | AD‡ | 1.04 ± 0.03 | 1.06 ± 0.04 | .217 |
|  | RD‡ | 0.63 ± 0.03 | 0.63 ± 0.02 | .428 |
| R DLPFC-Striatum | FA | 0.33 ± 0.02 | 0.32 ± 0.02 | .812 |
|  | MD‡ | 0.79 ± 0.02 | 0.81 ± 0.03 | .074 |
|  | AD‡ | 1.07 ± 0.03 | 1.09 ± 0.04 | .061 |
|  | RD‡ | 0.65 ± 0.02 | 0.67 ± 0.04 | .155 |

Abbreviations: AD, axial diffusivity; DLPFC, dorsolateral prefrontal cortex; FA, fractional anisotropy; L, left; MD, mean diffusivity; OCD, obsessive-compulsive disorder; R, right; RD, radial diffusivity

**†** Analysis of covariance controlling for age and gender effects.

‡ units = × 10^-3^mm^2^/s
