# Supplementary material for: Altered Fronto-Striatal Fiber Topography and Connectivity in Obsessive-Compulsive Disorder
Source: PLoS One. 2014 Nov 6;9(11):e112075. doi: 10.1371/journal.pone.0112075 (PMC4222976; doi:10.1371/journal.pone.0112075)
Supplement: Table S3 — Results of multiple regression analyses predicting each DWI index from the Y-BOCS, HDRS and HARS total scores in patients with OCD (N = 20, df = 16). (DOC) [file pone.0112075.s005.doc]

**Table S3. Results of multiple regression analyses predicting each DWI index from the Y-BOCS, HDRS and HARS total scores in patients with OCD (N = 20, *df* = 16)**

| Dependent variables | | Independent variables | Coefficients | | |
| --- | --- | --- | --- | --- | --- |
| Fibers | Diffusion indices |  | B | *t* | *P* |
| L OFC-Striatum | FA | Y-BOCS | -.001 | -1.215 | .242 |
|  |  | HDRS | -.002 | -.674 | .510 |
|  |  | HARS | -.001 | -.375 | .713 |
|  | MD | Y-BOCS | .000 | -.225 | .825 |
|  |  | HDRS | .000 | -.686 | .503 |
|  |  | HARS | 7.067E-007 | .332 | .744 |
|  | AD | Y-BOCS | .000 | -1.136 | .273 |
|  |  | HDRS | .000 | -1.080 | .296 |
|  |  | HARS | 6.989E-007 | .214 | .833 |
|  | RD | Y-BOCS | 6.192E-007 | .453 | .657 |
|  |  | HDRS | .000 | -.170 | .867 |
|  |  | HARS | 7.024E-007 | .290 | .775 |
| L DLPFC-Striatum | FA | Y-BOCS | .000 | -.435 | .669 |
|  |  | HDRS | -.002 | -.810 | .430 |
|  |  | HARS | .001 | .820 | .424 |
|  | MD | Y-BOCS | 1.561E-007 | .148 | .884 |
|  |  | HDRS | 2.015E-006 | .713 | .486 |
|  |  | HARS | .000 | -1.189 | .252 |
|  | AD | Y-BOCS | .000 | -.120 | .906 |
|  |  | HDRS | 4.603E-007 | .105 | .917 |
|  |  | HARS | .000 | -.705 | .491 |
|  | RD | Y-BOCS | 3.203E-007 | .289 | .777 |
|  |  | HDRS | 2.760E-006 | .928 | .367 |
|  |  | HARS | .000 | -1.163 | .262 |
| L dACC-Striatum | FA | Y-BOCS | -.001 | -.508 | .619 |
|  |  | HDRS | -.003 | -.727 | .478 |
|  |  | HARS | .003 | .927 | .368 |
|  | MD | Y-BOCS | 1.353E-006 | 1.818 | .088 |
|  |  | HDRS | .000 | -.434 | .670 |
|  |  | HARS | .000 | -1.296 | .213 |
|  | AD | Y-BOCS | 1.085E-006 | .823 | .423 |
|  |  | HDRS | .000 | -1.491 | .155 |
|  |  | HARS | 2.844E-007 | .122 | .904 |
|  | RD | Y-BOCS | 1.447E-006 | .941 | .361 |
|  |  | HDRS | 1.433E-006 | .347 | .733 |
|  |  | HARS | .000 | -1.017 | .324 |
| R OFC-Striatum | FA | Y-BOCS | -.001 | -.658 | .520 |
|  |  | HDRS | -.005 | -1.238 | .233 |
|  |  | HARS | .003 | 1.332 | .202 |
|  | MD | Y-BOCS | 2.212E-006 | 1.169 | .259 |
|  |  | HDRS | 1.256E-007 | .025 | .981 |
|  |  | HARS | .000 | -.112 | .912 |
|  | AD | Y-BOCS | 1.973E-006 | .788 | .442 |
|  |  | HDRS | .000 | -.816 | .426 |
|  |  | HARS | 3.305E-006 | .746 | .466 |
|  | RD | Y-BOCS | 2.314E-006 | 1.178 | .256 |
|  |  | HDRS | 2.889E-006 | .548 | .591 |
|  |  | HARS | .000 | -.633 | .536 |
| R DLPFC-Striatum | FA | Y-BOCS | .000 | -.209 | .837 |
|  |  | HDRS | -.004 | -1.893 | .077 |
|  |  | HARS | .002 | 1.493 | .155 |
|  | MD | Y-BOCS | 7.889E-007 | . 516 | .613 |
|  |  | HDRS | 3.334E-006 | .813 | .428 |
|  |  | HARS | .000 | -.123 | .904 |
|  | AD | Y-BOCS | 6.903E-007 | .372 | .715 |
|  |  | HDRS | .000 | -.203 | .842 |
|  |  | HARS | 2.050E-006 | .624 | .541 |
|  | RD | Y-BOCS | 8.336E-007 | .537 | .599 |
|  |  | HDRS | 5.514E-006 | 1.324 | .204 |
|  |  | HARS | .000 | -.565 | .580 |
| R dACC-Striatum | FA | Y-BOCS | 5.650E-005 | .049 | .962 |
|  |  | HDRS | -.001 | -.195 | .847 |
|  |  | HARS | .000 | -.183 | .857 |
|  | MD | Y-BOCS | 5.117E-007 | .436 | .669 |
|  |  | HDRS | .000 | -.742 | .469 |
|  |  | HARS | 6.400E-007 | .308 | .762 |
|  | AD | Y-BOCS | 8.146E-007 | .495 | .627 |
|  |  | HDRS | .000 | -.532 | .602 |
|  |  | HARS | .000 | -.148 | .884 |
|  | RD | Y-BOCS | 3.304E-007 | .245 | .810 |
|  |  | HDRS | .000 | -.628 | .539 |
|  |  | HARS | 1.107E-006 | .464 | .649 |
