# Supplementary material for: Altered Fronto-Striatal Fiber Topography and Connectivity in Obsessive-Compulsive Disorder
Source: PLoS One. 2014 Nov 6;9(11):e112075. doi: 10.1371/journal.pone.0112075 (PMC4222976; doi:10.1371/journal.pone.0112075)
Supplement: Table S4 — Z -component (dorsal-ventral) coordinates of center-of-gravity (COG) for each connectivity map in the MNI152 standard space controlling for past medication effects. (DOC) [file pone.0112075.s006.doc]

**Table S4.** *Z*-component (dorsal-ventral) coordinates of center-of-gravity (COG) for each connectivity map in the MNI152 standard space controlling for past medication effects

|  |  | ***Z*-component coordinate of COG (mm) Mean (SD)** | |  |
| --- | --- | --- | --- | --- |
| **Seed ROI** | **Target ROI** | **Healthy Controls (n = 20)** | **Patients with OCD (n = 20)** | ***P* value†** |
| Left striatum | OFC | -6.21 ± 1.71 | -5.08 ± 1.71 | .066 |
|  | DLPFC | 1.86 ± 2.78 | 1.48 ± 2.16 | .972 |
|  | dACC | 7.11 ± 2.33 | 7.41 ± 1.92 | .626 |
| Right striatum | OFC | -6.20 ± 1.67 | -5.21 ± 1.78 | .044* |
|  | DLPFC | 3.14 ± 2.64 | 3.25 ± 3.01 | .158 |
|  | dACC | 10.05 ± 4.05 | 9.60 ± 4.56 | .885 |

Abbreviations: dACC, dorsal anterior cingulate cortex; DLPFC, dorsolateral prefrontal cortex; OCD, obsessive-compulsive disorder; OFC, orbitofrontal cortex; ROI, region of interest

**†**Analysis of covariance controlling for age, gender, and past medication effects.

**P* < .05 (not corrected for multiple comparisons).
