# Supplementary material for: Altered Fronto-Striatal Fiber Topography and Connectivity in Obsessive-Compulsive Disorder
Source: PLoS One. 2014 Nov 6;9(11):e112075. doi: 10.1371/journal.pone.0112075 (PMC4222976; doi:10.1371/journal.pone.0112075)
Supplement: Table S5 — Diffusion indices of fibers between the OFC and the striatum controlling for past medication effects. (DOC) [file pone.0112075.s007.doc]

**Table S5.** Diffusion indices of fibers between the OFC and the striatum controlling for past medication effects

| **Fibers** | **Diffusion indices** | **Healthy Controls (n = 20)** | **Patients with OCD (n = 20)** | ***P* value†** |
| --- | --- | --- | --- | --- |
| L OFC-Striatum | FA | 0.32 ± 0.03 | 0.34 ± 0.03 | .012* |
|  | MD‡ | 0.81 ± 0.03 | 0.79 ± 0.03 | .189 |
|  | AD‡ | 1.11 ± 0.06 | 1.11 ± 0.05 | .914 |
|  | RD‡ | 0.66 ± 0.04 | 0.64 ± 0.03 | .039* |
| R OFC-Striatum | FA | 0.32 ± 0.02 | 0.34 ± 0.03 | .061 |
|  | MD‡ | 0.80 ± 0.03 | 0.81 ± 0.04 | .731 |
|  | AD‡ | 1.09 ± 0.05 | 1.12 ± 0.06 | .133 |
|  | RD‡ | 0.66 ± 0.04 | 0.66 ± 0.05 | .214 |

Abbreviations: AD, axial diffusivity; FA, fractional anisotropy; L, left; MD, mean diffusivity; OCD, obsessive-compulsive disorder; OFC, orbitofrontal cortex; R, right; RD, radial diffusivity

**†** Analysis of covariance controlling for age, gender, and past medication effects.

**P* < .05 (not corrected for multiple comparisons).

‡ units = × 10^-3^mm^2^/s
