# Supplementary material for: Altered Fronto-Striatal Fiber Topography and Connectivity in Obsessive-Compulsive Disorder
Source: PLoS One. 2014 Nov 6;9(11):e112075. doi: 10.1371/journal.pone.0112075 (PMC4222976; doi:10.1371/journal.pone.0112075)
Supplement: Table S7 — Diffusion indices of fibers between the dACC and the striatum controlling for past medication effects. (DOC) [file pone.0112075.s009.doc]

**Table S7.** Diffusion indices of fibers between the dACC and the striatum controlling for past medication effects

| **Fibers** | **Diffusion indices** | **Healthy Controls (n = 20)** | **Patients with OCD (n = 20)** | ***P* value†** |
| --- | --- | --- | --- | --- |
| L dACC-Striatum | FA | 0.37 ± 0.04 | 0.38 ± 0.04 | .365 |
|  | MD‡ | 0.77 ± 0.08 | 0.75 ± 0.02 | .385 |
|  | AD‡ | 1.08 ± 0.09 | 1.06 ± 0.03 | .820 |
|  | RD‡ | 0.62 ± 0.08 | 0.59 ± 0.04 | .299 |
| R dACC-Striatum | FA | 0.35 ± 0.03 | 0.36 ± 0.03 | .063 |
|  | MD‡ | 0.77 ± 0.02 | 0.76 ± 0.03 | .482 |
|  | AD‡ | 1.06 ± 0.03 | 1.07 ± 0.04 | .388 |
|  | RD‡ | 0.63 ± 0.03 | 0.61 ± 0.03 | .202 |

Abbreviations: AD, axial diffusivity; dACC, dorsal anterior cingulate cortex; FA, fractional anisotropy; L, left; MD, mean diffusivity; OCD, obsessive-compulsive disorder; R, right; RD, radial diffusivity

**†** Analysis of covariance controlling for age, gender, and past medication effects.

‡ units = × 10^-3^mm^2^/s
